# Case Report: Ruptured Middle Cerebral Artery Aneurysm With Intrasylvian Hematoma Successfully Treated by Coil Embolization and Minimally Invasive Puncture and Drainage

**DOI:** 10.3389/fneur.2021.646990

**Published:** 2021-06-21

**Authors:** Zhen Li, Quan Hu, Li Zhao, Huayun Huang, Shizhong Zhang, Limin Zheng, Guojun Wang

**Affiliations:** ^1^Department of Neurosurgery, Taian Central Hospital, Taian, China; ^2^Color Ultrasonic Room, Taian Central Hospital, Taian, China

**Keywords:** ruptured middle cerebral artery aneurysm, hematoma, coil embolization, minimally invasive puncture and drainage, outcomes

## Abstract

Up to one-third (12–35%) of patients with aneurysmal subarachnoid hemorrhage experience intracerebral hematoma. Ruptured middle cerebral artery (MCA) aneurysm with hematoma is usually accompanied by progressive cerebral swelling with poor outcomes; however, it can be successfully treated by coil embolization and minimally invasive puncture and drainage. From February 2012 to March 2019, six surgeries for ruptured MCA aneurysms with intrasylvian hematoma were performed at our clinic. All patients had intracranial hematomas of <30 ml and GCS scores >8. The patients were treated by coil embolization and minimally invasive puncture and drainage. The aneurysms in all patients were completely embolized and the hematomas were mostly removed by minimally invasive puncture. The Glasgow outcome scale (GOS) scores of all patients were more than 4 at discharge when they discharged. Coil embolization and minimally invasive puncture and drainage are viable treatments for ruptured MCA aneurysms with hematomas, especially if the patient is too old, in a complicated state to undergo craniotomy, is unwilling to undergo craniotomy, or is at a greater risk of bleeding 3 days after surgery.

## Introduction

Intracerebral hematoma (ICH) due to rupture of intracranial aneurysm (IA) occurs in 10–38% of cases with subarachnoid hemorrhage (SAH) (1). Aneurysmal ICH complicates the natural course of the disease and is associated with increased morbidity and mortality (2). Middle cerebral artery (MCA) aneurysms are most likely to result in an intracerebral and intrasylvian hematoma after their rupture (3, 4). About 30-37.5% of ruptured middle cerebral artery aneurysms can lead to intracranial hematoma. Most neurosurgeons prefer microsurgical clipping for treatment of such patients. Smith et al., through a systematic review and meta-analysis, recommended surgical clipping for unruptured MCA aneurysms (5). Along with the publication of International Subarachnoid Aneurysm Trial (ISAT) and the improvement of neurointervention techniques, more neurosurgical centers have been opting for endovascular coiling (6, 7). However, for ruptured MCA aneurysms with hematomas, most patients still prefer craniotomy clipping. In China, some scholars believe that endovascular coiling is effective in patients with ruptured MCAs (8). Whether interventional embolization can be performed for ruptured middle cerebral artery aneurysms with hematoma is still uncertain. We have reported six cases with ruptured MCA aneurysm and intrasylvian hematoma that were successfully treated by coil embolization and minimally invasive puncture and drainage.

## Subjects and Methods

### Patient Population

From February 2012 to March 2019, six patients (total of six MCA aneurysms) underwent endovascular coiling and minimally invasive puncture and drainage in our institution. The clinical condition at admission was classified according to the Hunt–Hess grade. Post-operative CT scans were obtained for all patients, and the hematoma volume was calculated using the formula a × b × c/2. The clinical outcomes were graded according to the Glasgow Outcome Scale (GOS). MCA aneurysms were confirmed by DSA and treated by an endovascular approach. All the intracranial hematomas were confirmed by CT and treated by minimally invasive puncture and drainage (Table 1).

**Table 1:** Characteristics of six patients with middle cerebral artery aneurysms.

	**Sex**	**Age**	**GCS**	**Hunt-hess**	**GOS**	**Antiplatelet**	**Stent**
Case 1	Male	42	11	3	5	NO	NO
Case 2	Male	48	9	4	4	NO	NO
Case 3	Female	69	10	2	5	YES	YES
Case 4	Female	44	12	2	5	NO	NO
Case 5	Female	76	10	3	4	NO	NO
Case 6	Male	50	13	2	5	YES	YES

*GCS, Glasgow coma scale; GOS, Glasgow outcome scale*.

The outcomes of patients were evaluated by GOS. Patients with GOS score 1–3 were defined as having poor outcomes and those with GOS scores 4–5 were defined as having good outcomes. Informed consent was obtained from patients and the study was approved by our institutional review board.

## Results

### Baseline Characteristics

The clinical characteristics and treatment details of the six patients are shown in Table 1. The sample comprised two men and four women with a mean age of 58.7 (range 42–76) years. All patients had a GLS score >8 before the surgery. The GOS scores of all patients were more than 4 at discharge.

### Illustrative Cases

#### Case One

**Figure 1:**
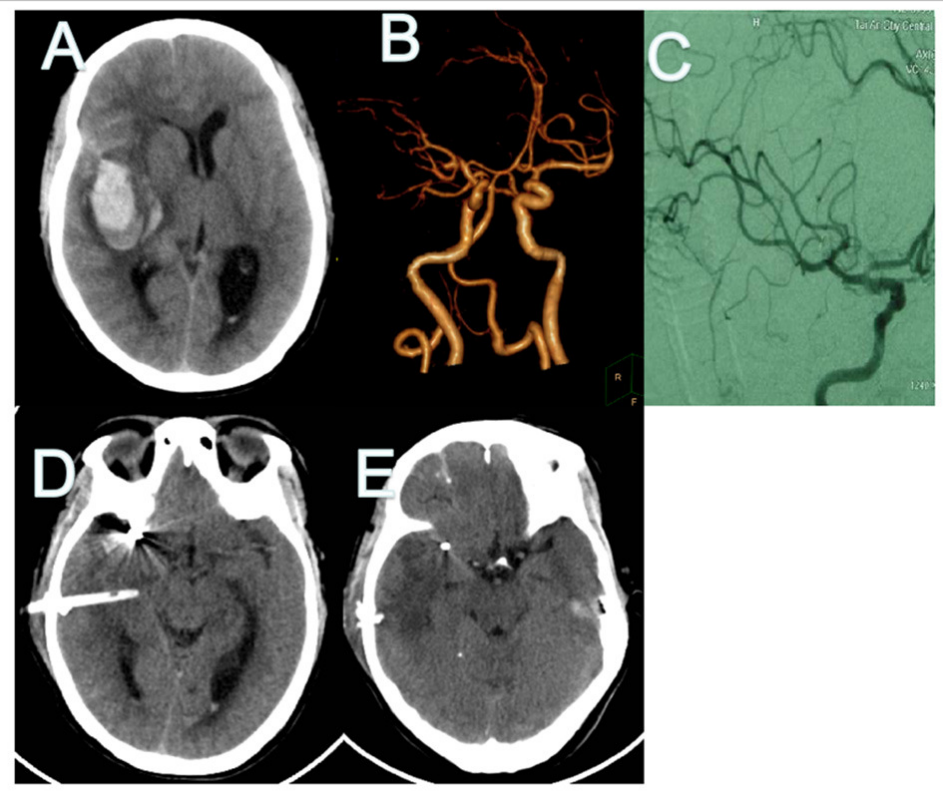
CT and CT angiography in case 1. **(A,B)** Initial CT scans reveal characteristic findings of ruptured MCA aneurysm with intrasylvian hematoma. Hematoma volume was calculated as 28.8 ml. **(C)** Post-operative angiography shows the aneurysm is completely occluded and parent artery is unimpeded. **(D)** Immediate post-operative CT finding the drainage tube was in good position. **(E)** CT finding Hematoma's almost drained clean on POD 2. CT, computed tomography; MCA, middle cerebral artery; POD, post-operative day.

A 48-year-old man presented to the emergency room with a severe headache after sudden loss of consciousness. Initial consciousness level was deep drowsy (Hunt–Hess 4). Brain CT showed a typical SAH from MCA aneurysmal rupture, in which the SAH was mainly dispersed prominently along the left Sylvian fissure. Hematoma volume was calculated as 28.7 ml using the formula a × b × c/2 (Figures 1A,B). Coil embolization surgery was performed 6 h after admission. Minimally invasive puncture and drainage were performed immediately after the operation. Surgery was performed successfully on the ruptured aneurysm and no complication was observed during the procedure (Figures 1C,D). On the two day after the operation, CT showed that the hematoma had disappeared (Figure 1E).

#### Case Two

**Figure 2:**
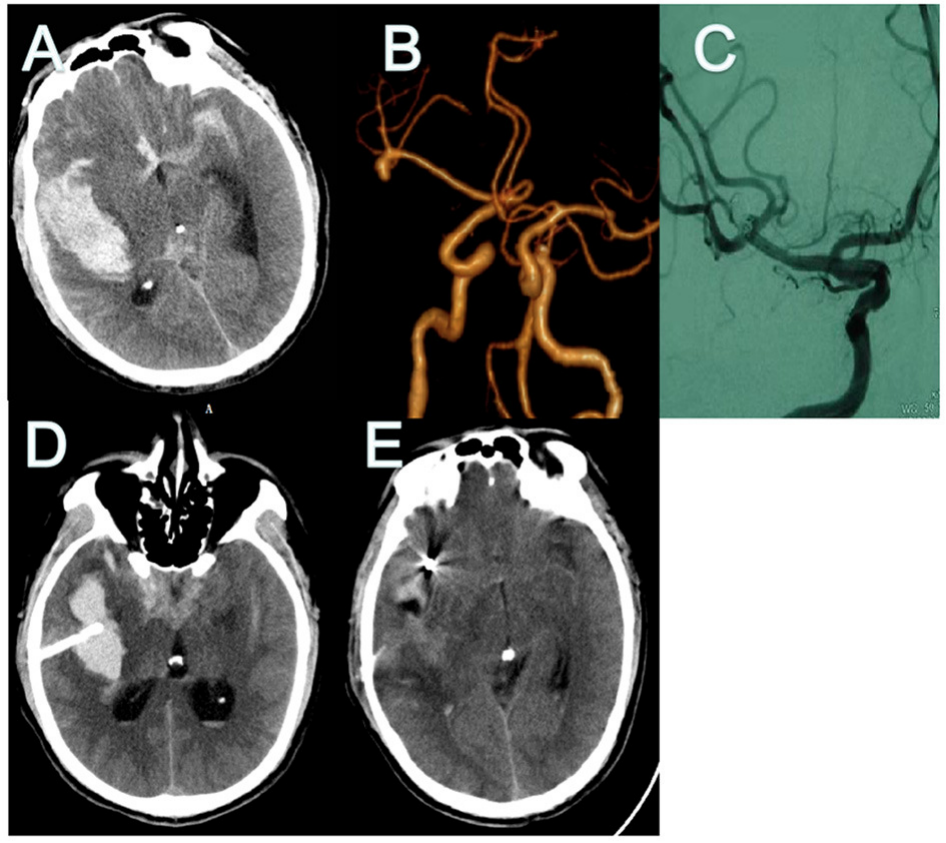
CT and CT angiography in case 1. **(A,B)** Initial CT scans reveal characteristic findings of ruptured MCA aneurysm with intrasylvian hematoma. Hematoma volume was calculated as 16.5 ml. **(C)** Post-operative angiography shows the aneurysm is completely occluded and parent artery is unimpeded. **(D)** Immediate post-operative CT finding the drainage tube was in good position. **(E)** CT finding Hematoma's almost drained clean on POD 3. CT, computed tomography; MCA, middle cerebral artery; POD, post-operative day.

A 42-year-old man was transferred to our hospital 3 days after the onset of symptoms (a severe headache) (Hunt-Hess 2). Brain CT and CT angiography revealed SAH from ruptured MCA aneurysm and large amounts of intrasylvian hematoma (about 16.5 ml; Figures 2A,B). Coil embolization surgery was performed 5 h after admission. Minimally invasive puncture and drainage were performed immediately after the operation. Surgery was performed successfully on the ruptured aneurysm and there was no complication during the procedure (Figures 2C,D). On the third day after the operation, CT showed that the hematoma had almost disappeared (Figure 2E).

## Discussion

The MCA has a complex anatomical structure, which contains multiple ventral perforating arteries. It is widely distributed, with branches lacking collagen circulation. Ruptured aneurysms in the brain can lead to neurological disorders, such as hemiplegia, aphasia, and paresthesia. MCA aneurysms account for 20% of intracranial aneurysms (1). Ruptured intracranial aneurysms are often accompanied by large intracranial hematomas, as compared to other intracranial aneurysms. Hence, the Hunt–Hess grade is worse in such cases. About 30% of ruptured middle cerebral artery aneurysms can lead to intracranial hematoma (7). Zhao et al. reported that 37.3% of the patients had intracranial hematomas, and that inappropriate treatment may affect branch blood flow causing chemical syndrome or cerebral infarction (8).

Currently, the treatment methods for ruptured aneurysms mainly include microsurgical clipping and endovascular embolization. With the publication of ISAT, more neurosurgical centers begun to choose endovascular embolization. However, they have not elaborated on the best treatment for aneurysms, which remains controversial. The complex anatomical structure of the MCA, such as bifurcation and multiple branches, increases the difficulty of performing an interventional surgery. For ruptured wide-necked aneurysms, early stent placement can lead to thrombosis (5, 9). However, for some patients who are not suitable for craniotomy (such as old age, long-term anticoagulant therapy, etc.) there is still no good treatment plan.

With the improvement of interventional techniques and the application of new interventional materials, endovascular embolization techniques, especially three-dimensional rotary angiography, and stent-assisted embolization, can be used to treat more complex large aneurysms. Three-dimensional rotational angiography can clearly show the anatomical features of the MCA and its branches. Stenting reduces the recurrence rate of MCA aneurysms by changing the cerebral hemodynamics and endothelialization of the stent. The effect of stent network on the parent artery branch remains minor. Many studies have confirmed the safety and efficacy of endovascular embolization and have obtained comparable results to that of surgical clipping (2, 5, 10, 11). At present, most scholars still believe that microsurgical clipping should be the preferred treatment for ruptured MCA aneurysms with hematoma. Our surgical experience was that the brain tissue was obviously swollen and the lateral fissure was difficult to separate after 3 days of subarachnoid hemorrhage. We reported six patients with ruptured MCA aneurysms with intrasylvian hematoma who were treated in our clinic. All patients had intracranial hematomas of <30 ml and GCS scores >8. The patients were treated by coil embolization and minimally invasive puncture and drainage. Good prognosis was obtained. However, microsurgical clipping remains to be the preferred treatment for patients with massive hematomas and cerebral hernia.

There were some limitations in this study. This was not a randomized study, and the number of patients and the duration of follow-up were limited. Due to the small sample and lack of randomized controlled studies, our retrospective study lacked the support of a statistical analysis, However, this paper is only published as a case report and only provides a treatment idea. For the advantages and disadvantages of this approach, a lot of comprehensive analysis of clinical cases is needed, which is our work in the future.

## Conclusion

Coil embolization and minimally invasive puncture and drainage are viable treatments for ruptured MCA aneurysms with hematomas, especially if the patient is too old, in a complicated condition to undergo craniotomy, is unwilling to undergo craniotomy, or is at a greater risk of bleeding 3 days after surgery. In addition, for some young women or special working population, the future work might be affected by surgical scar.

## Data Availability Statement

The original contributions presented in the study are included in the article/supplementary material, further inquiries can be directed to the corresponding author/s.

## Ethics Statement

The studies involving human participants were reviewed and approved by the ethics committee of Taian Central Hospital. The patients/participants provided their written informed consent to participate in this study. Written informed consent was obtained from the individual(s) for the publication of any potentially identifiable images or data included in this article.

## Author Contributions

All authors listed have made a substantial, direct and intellectual contribution to the work, and approved it for publication.

## Conflict of Interest

The authors declare that the research was conducted in the absence of any commercial or financial relationships that could be construed as a potential conflict of interest.
